# Orthopnea secondary to brachial plexitis with bilateral diaphragmatic paralysis

**DOI:** 10.1186/s12890-023-02828-3

**Published:** 2024-01-12

**Authors:** Mohamad El Labban, Philippe R. Bauer

**Affiliations:** 1 Department of Internal Medicine, Mayo Clinic Health System, 101 Martin Luther King Dr, Mankato, MN USA; 2 Pulmonary and Critical Care Medicine, Mayo Clinic, Rochester, MN USA

**Keywords:** Orthopnea, Diaphragmatic paralysis, Pulmonary function test, Parsonage-turner syndrome, Brachial plexopathy

## Abstract

**Background:**

Diaphragmatic paralysis can present with orthopnea. We report a unique presentation of bilateral diaphragmatic paralysis, an uncommon diagnosis secondary to an unusual cause, brachial plexitis. This report thoroughly describes the patient’s presentation, workup, management, and outcome. It also reviews the literature on diaphragmatic paralysis and Parsonage-Turner syndrome.

**Case presentation:**

A 50-year-old male patient developed insidious orthopnea associated with left shoulder and neck pain over three months with no associated symptoms. On examination, marked dyspnea was observed when the patient was asked to lie down; breath sounds were present and symmetrical, and the neurological examination was normal. The chest radiograph showed an elevated right hemidiaphragm. Echocardiogram was normal. There was a 63% positional reduction in Forced Vital Capacity and maximal inspiratory and expiratory pressures on pulmonary function testing. The electromyogram was consistent with neuromuscular weakness involving both brachial plexus and diaphragmatic muscle (Parsonage and Turner syndrome).

**Conclusions:**

Compared to unilateral, bilateral diaphragmatic paralysis may be more challenging to diagnose. On PFT, reduced maximal respiratory pressures, especially the maximal inspiratory pressure, are suggestive. Parsonage-Turner syndrome is rare, usually with unilateral diaphragmatic paralysis, but bilateral cases have been reported.

## Background

Diaphragmatic paralysis is uncommon and can be unilateral or bilateral. The most common cause of paralysis, usually unilateral, is phrenic nerve injury due to cardiothoracic surgery [1]. Cervical spine (C3-C5) abnormalities such as cervical compressive tumors, cervical spondylosis, and neck trauma are also associated with phrenic nerve injury [2, 3]. Less commonly, phrenic nerve involvement in the setting of brachial plexopathy can cause diaphragmatic paralysis. Parsonage and Turner were the first to well describe neuralgic amyotrophy in 1948 as an idiopathic disorder characterized as pain with associated muscular weakness in the distribution of single or multiple motor nerves [4]. Nowadays, neuralgic amyotrophy is regarded as an inflammatory disorder of the brachial plexus or “plexitis” [5]. The classic presentation of this syndrome includes the onset of severe pain followed by weakness in the muscles innervated by the brachial plexus. Atypically and less often, the syndrome may involve nerves outside the brachial plexus, including the phrenic nerve (only around 2% of idiopathic neuralgic amyotrophy [6, 7]. We present a unique and subtle presentation of brachial plexopathy leading to bilateral diaphragmatic paralysis.

## Case description

A 50-year-old male patient presented to the clinic complaining of shortness of breath for three months duration that started insidiously. It occurred only in a supine position, with the patient specifying: “I can’t lay flat for a second.” He has been having difficulties sleeping despite propping up two to three pillows. There was no dyspnea at rest, the patient could exercise three times a week without limitation, and there were no episodes of nocturnal dyspnea. There was no cough, postnasal drip, reflux symptoms, or chest pain. There was no recent upper respiratory illness, but the patient reported intermittent episodes of severe neck and left shoulder pain for the past three months that occurred without apparent trauma and did not improve with positional changes, muscle relaxants, and over-the-counter pain relievers. This pain had worsened gradually over time. There was no associated left upper extremity numbness, weakness, or atrophic changes. Family history was unknown because the patient was adopted. Past medical history was remarkable for primary hypertension and obesity class I with a body mass index of 32 and no surgery or trauma. The patient was a former smoker (8 pack-year) who quit…years ago.

The patient appeared well on examination, with a pulse of 85 beats/min, blood pressure of 179/105 mm Hg, temperature of 36.6 °C, and oxygen saturation of 98% while breathing ambient air. The patient had good bilateral breathing sounds without wheeze, rhonchi or, or crackle in a seated position. There was good expansion of the chest wall. The patient did not tolerate lying flat, immediately gasping for air, and sitting back up immediately. Abdomen was nondistended. There was mild bilateral pitting edema of the lower extremities. There was no limitation of motion or tenderness with passive and active neck and left shoulder movement. Peripheral pulses were all present and symmetrical. Neurological examination was normal that included motor, sensory, cranial nerves, coordination, and gait testing. Further neurological examination of the bilateral upper extremities revealed normal deep tendon reflexes with the absence of spasticity and weakness. The remainder of the exam was unremarkable.

Blood work (reference ranges in parenthesis) showed hemoglobin of 17.8 g/dL (11.6 to 15.0 g/dL). Leukocytes 6.3 × 109/L (3.4 to 9.6 × 109/L), platelets 237 × 10^9^/L (157 to 371 × 10^9^/L), creatinine 0.82 mg/dL (0.59 to 1.04 mg/dL), Aspartate Aminotransferase 73 (8–48 U/L); Alanine Aminotransferase 127 (7–55 U/L), C-reactive protein < 3.0 mg/L (≤ 8.0 mg/L), Troponin T, 9 mg/L (≤ 15 ng/L), NT-Pro Brain-Type Natriuretic Peptide 35 (≤ 87 pg/ml), and D-Dimer 271 (≤ 500 ng/ml).

**Fig. 1:**
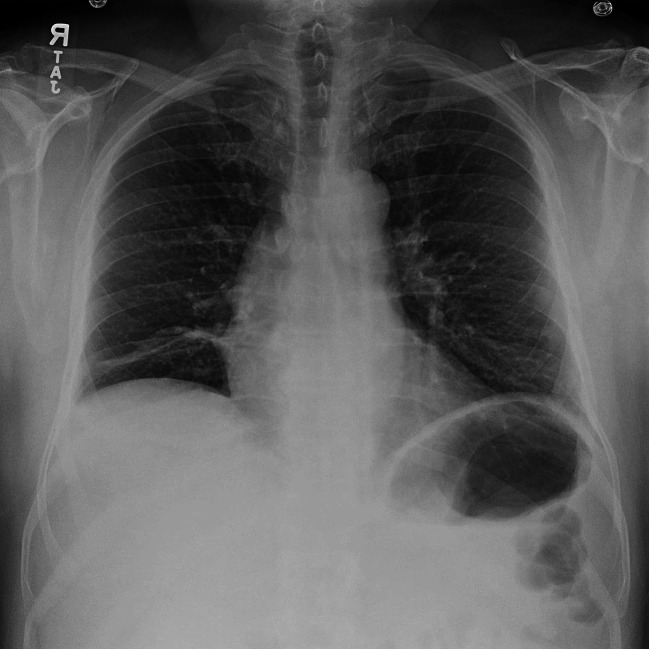
Chest radiograph showing elevated right hemidiaphragm

The chest radiograph (Fig. 1) showed right basilar atelectasis and elevated right hemidiaphragm. The echocardiogram showed an Ejection Fraction of 60%, no wall motion abnormality, normal left ventricular diastolic function, and a trileaflet aortic valve with mild regurgitation. The right ventricle was normal in size and function. The right ventricular systolic pressure could not be obtained. Given the presentation of situational orthopnea, respiratory muscle weakness was suspected. A Sniff test showed subtle symmetrical decreased respiratory motion of both hemi-diaphragms.

**Fig. 2:**
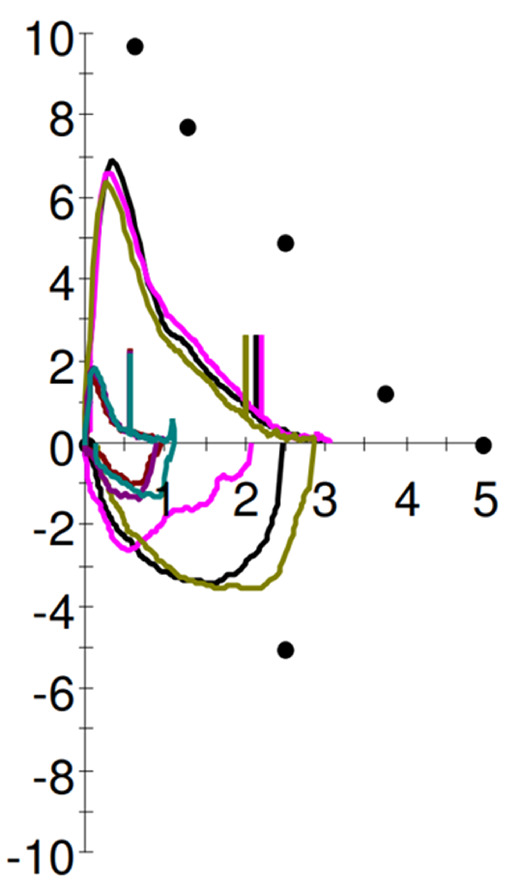
Flow-volume loop. Blue arrow: three attempts in the upright position. Orange arrow: three attempts in a supine position. Green arrow: FEV1 in the upright position. Black arrow: FEV1 in the supine position

Pulmonary function testing (Table 1; Fig. 2) showed a forced vital capacity (FVC) of 3,06 L (61% of predicted) while sitting and 1.11 L (22%) while supine, a 63% positional change. Both maximal inspiratory and expiratory pressures were reduced.

**Table 1:** Pulmonary function testing

	Sitting				Supine		
	Actual	Predicted	% Predicted	Lower Limit Normal	Actual	% Predicted	% Change
FVC (L)	**3.06**	4.93	**61**	3.86	**1.11**	**22**	**-63**
FEV1 (L)	**2.22**	3.89	**57**	3.04	**0.59**	**15**	**-73**
FEV1/FVC (%)	72.69	79.15	91	68.23	52.72	66	-27
FEF 25–75% (L/sec)	1.51	3.55	42	1.91	0.19	5	-87
FEF Max (L/sec)	6.88	9.76	70	7.45	1.87	19	-72
MIP (cmH2O)	-41	-123	**33**				
MEP (cmH2O)	135	233	**57**				

FVC: Forced vital capacity; FEV1: Forced expiratory volume in 1 s; FEF: Forced expiratory flow; MIP: Maximal inspiratory pressure; MEP: Maximal expiratory pressure. FVC and FEV1 values marked in bold to highlight the significant change sitting vs. supine.

The reduced maximal respiratory pressures and change in supine forced vital capacity was consistent with diaphragmatic weakness and/or paralysis, and an underlying neuromuscular disorder needed to be assessed further. Because of the neck pain and bilateral diaphragmatic weakness, an ultrasound and an electromyography (EMG) of the diaphragm was performed. The ultrasound showed reduced diaphragm thickness bilaterally. The nerve conduction studies showed absent bilateral phrenic responses. The EMG confirmed the diagnosis of bilateral diaphragm paralysis and large polyphasic motor units in primarily C5-innervated muscle, suggesting an inflammatory brachial plexitis. The patient underwent Computed Tomography (CT) of the neck that did not reveal any cervical spine trauma, mass, or significant stenosis. Further workup to rule out paraneoplastic neuropathy, postinfectious neuropathy, and connective tissue diseases (Dermatomyositis, Polymyositis, mixed connective tissue disease, Systemic Lupus Erythematosus) was negative, including a full autoimmune panel (rheumatological and neurological). The final diagnosis was bilateral diaphragmatic paralysis secondary to idiopathic neuralgic amyotrophy of the brachial plexus with bilateral phrenic nerve involvement consistent with Parsonage and Turner syndrome. A brachial plexus MRI could not be obtained because the patient couldn’t lie flat. Instead, an empirical 12-week intravenous Methylprednisolone treatment was initiated. The patient received 1 gram of Methylprednisolone daily for 3 days, then weekly for five weeks, and then every two for three doses. On follow-up two months later, symptoms had markedly improved. The patient was now able to lie flat using only one pillow. He felt relieved that he could now lie down without “suffocating.” Four months after the last methylprednisolone dose, a CT chest done showed no evidence of no suspicious pulmonary masses, nodules, or adenopathy.

## Discussion and conclusions

Shortness of breath triggered or exacerbated in a supine position defines orthopnea. A broad differential of diagnoses can cause orthopnea. The most common cause of orthopnea is elevated pulmonary capillary pressure, with pulmonary edema due to left-sided heart failure. Other causes include reduction of the functional residual capacity, as in the case of central obesity, tense ascites, or large pleural effusion., and neuro-muscular disorders such as amyotrophic lateral sclerosis, Myasthenia Gravis, or diaphragmatic paralysis. The diaphragm is the primary inspiratory muscle; the sternomastoid, trapezius, latissimus dorsi, and pectoralis minor and major also participate to inspiration. The diaphragm is innervated by the phrenic nerve, derived from the third, fourth, and fifth cervical nerve roots.

Diagnostic evaluation of suspected diaphragm paralysis starts with a comprehensive history and physical examination. After inquiring about positional dyspnea, further evaluation should include asking about the history of childhood poliomyelitis, infections including Lyme, syphilis, and infection with human immunodeficiency viruses [8], remote history of neck or cardiothoracic surgery or trauma, history of associated neurological symptoms such as muscle weakness or numbness. The physical examination can be misleading; however, specific findings can help support potential causes. Upper extremities weakness or numbness in cases of brachial plexus or cervical spine pathology, lymphadenopathy in cases of underlying occult tumors, and scars of previous surgeries. The principal causes of diaphragmatic weakness are further mentioned in Table 2.

**Table 2:** Principal causes of diaphragmatic paralysis [9]

Causes	Unilateral Diaphragmatic Paralysis	Bilateral Diaphragmatic Paralysis
Idiopathic	X	X
Amyotrophic Lateral Sclerosis		X
Complete cervical spinal cord dysfunction		X
Partial cervical spinal cord dysfunction	X	X
Critical illness myopathy/neuropathy		X
Guillain-Barré syndrome		X
Multiple Sclerosis		X
Phrenic nerve dysfunction		
Blunt trauma	X	
Brachial plexopathy	X	X
Post cardiac surgery	X	
Idiopathic neuropathy	X	X
Paraneoplastic neuropathy		X
Postinfectious neuropathy	X	X
Radiation therapy	X	X
Dermatomyositis/ polymyositis		X
Inclusion body myositis		X
Systemic lupus erythematosus		X
Hyperthyroidism or hypothyroidism		X
Malnutrition	X	X

A chest radiograph showing elevated hemidiaphragm is sensitive but not specific for unilateral diaphragmatic paralysis and can be pseudo-normal in case of bilateral paralysis. In our patient, the chest radiograph did not display any elevated hemi-diaphragm or small lung volumes. The fluoroscopic sniff test is easy to perform and is the initial test of choice to diagnose diaphragmatic weakness. In cases of unilateral diaphragm paralysis, the sniff test is positive (absence of downward movement of the diaphragm) in over 90% of the cases [10]. In bilateral paralysis, however, the sniff test may be falsely negative. This happens because the accessory inspiratory muscles result in the upward movement of the ribs, resulting in the false appearance of the downward movement of the diaphragm [1, 11]. Spirometry findings may also be misleading. If respiratory muscle weakness is suspected, including maximal voluntary ventilation, maximal respiratory pressures, and supine-forced vital capacity should be ordered. The respiratory muscles’ contribution to tidal breathing varies depending on the body position. In an erect position, the intercostals can contribute around 30% to the tidal breath; however, their impact becomes significantly diminished in a supine position, and the diaphragm’s contribution goes over 90% [12]. In a study of 50 individuals with normal lung function, a delta FVC (difference between erect and supine) ≥ 25% in patients was an indicator of possible underlying diaphragm dysfunction [13]. In another study, a decrease in vital capacity ≥ 30% in the supine posture, compared to an erect posture, indicated bilateral diaphragm weakness [14]. In our patient, the positional drop in the FVC is secondary to absent bilateral phrenic nerve responses as per the nerve conduction study leading to bilateral diaphragmatic paralysis.

Patients with neuralgia amyotrophy should undergo routine blood work to rule out infectious causes such as human virus deficiency [8], Lyme disease, and syphilis, endocrine causes such as diabetes mellitus, hypothyroidism, and rheumatological causes such as myositis. Imaging of the cervical spine to rule out compressive tumors and spondylosis may be obtained. Regarding management, there is no specific treatment for neuralgic amyotrophy. There is no definite evidence that steroids are successful in treatment [7]. Physical therapy may help avoid muscle wasting but does not alter the course of the disease. The prognosis varies according to the different clinical forms of neuralgic amyotrophy. A study of 99 patients with brachial plexus neuropathy showed good recovery [15]. For patients with phrenic neuropathy, evidence suggests spontaneous recovery within two years with some residual symptoms [16].

This case report offers several lessons. First, symptomatic diaphragmatic paralysis is an uncommon diagnosis and is usually unilateral. Our patient had marked symptoms secondary to bilateral involvement. Second, the sniff test is a suitable screening method for diaphragmatic paralysis but can be misleading in cases of bilateral diaphragmatic involvement. Third, brachial plexopathy or plexitis is a rare cause of diaphragmatic paralysis, even more uncommon, causing bilateral involvement. The limitation of the study is the lack of follow-up of the pulmonary function testing glucocorticoid treatment for objective improvement of respiratory muscle weakness, although the clinical improvement was remarkable.

In conclusion, in the case of new-onset orthopnea, aside from assessing for heart failure, one needs to explore the possibility of diaphragmatic paralysis.

## Data Availability

Available upon request.

## Abbreviations

FVC: forced vital capacity
EMG: electromyography
CT: Computed Tomography
